# Direct-to-Consumer Genetic Testing in South Africa: Stumbling Over the First Legal Hurdle?

**DOI:** 10.17159/1727-3781/2022/v25i0a11764

**Published:** 2022-09-13

**Authors:** Amy Gooden, Donrich W Thaldar

**Affiliations:** University of KwaZulu-Natal South Africa; University of KwaZulu-Natal South Africa

**Keywords:** Direct-to-consumer genetic testing, saliva sample, removal, tissue, biological material, *National Health Act*, autonomy, privacy, human dignity, bodily integrity

## Abstract

Despite the growing popularity of direct-to-consumer genetic testing, there is minimal South African literature on the topic. The limited available research suggests that direct-to-consumer genetic testing is unregulated. However, we suggest that direct-to-consumer genetic testing is indeed regulated, and unusually so. The first step in the process – the collection of a saliva sample by consumers themselves – is unlawful on a plain reading of the *National Health Act* 61 of 2003 and the *Regulations Relating to the Use of Human Biological Material*. This is because these statutes require that certain healthcare professionals must remove saliva for genetic testing. Yet, on closer analysis, such an apparent ban on the self-collection of saliva is neither aligned with a purposive interpretation of the relevant legislation, nor would it survive constitutional scrutiny – as it impedes an individual’s autonomy. It is concluded that, contrary to a plain reading of the relevant statutes, individuals can lawfully collect their own saliva for direct-to-consumer genetic testing. To provide legal clarity we recommend that the relevant provisions of the *National Health Act* 61 of 2003 and *Regulations Relating to the Use of Human Biological* the *Material* be amended to allow individuals to collect their own saliva samples.

## 1 Introduction

The completion of the Human Genome Project^1^ and recent advances in science and technology have allowed genetic testing to be conducted inexpensively,^2^ expeditiously and directly by consumers. Individuals are thus able to access their genetic information without the intervention of a healthcare practitioner or genetic counsellor.^3^ Direct-to-consumer genetic testing refers to DNA tests for traits, medical or otherwise, that provide the communication and interpretation of results directly to consumers – thus bypassing healthcare professionals.^4^ However, recently the direct-to-consumer genetic testing model has changed, with more providers now requiring a physician’s involvement before and/or after testing.^5^

The internet has made the recent surge in direct-to-consumer genetic testing significantly easier by making such tests publicly accessible.^6^ Anyone with internet access and a credit card can purchase a test.^7^ Sample collection kits containing a buccal swab are mailed directly to consumers^8^ who then provide their saliva sample and return the sample via the post.^9^ Certain direct-to-consumer genetic testing providers offer a single test for one trait, others sell tests for a collection of traits, and some providers conduct genome-wide testing that analyses multiple genetic variants and provides results for numerous traits.^10^ Consumers can view the results of the genetic tests through their online accounts or on mobile apps.^11^ These accounts and apps offer personalised details and risk estimates of their susceptibility to certain genetic conditions or traits.^12^

The proliferation of direct-to-consumer genetic testing has generated discussion of the ethical, legal and social issues involved,^13^ the potential harms to consumers, as well as concerns about the commercialisation of genetic testing.^14^ While direct-to-consumer genetic testing holds several advantages, including patient autonomy and empowerment,^15^ involvement in healthcare decisions,^16^ increased affordability and speed,^17^ greater privacy and convenience,^18^ and enhanced genetic literacy^19^ – the field remains contentious. The questionable validity, accuracy and utility of tests, the absence of healthcare professional involvement, the potential misinterpretation of results, privacy concerns about the use and confidentiality of the genetic data,^20^ follow-up costs which burden healthcare systems,^21^ genetic discrimination and exaggerated advertising and marketing claims have all been raised as legal and ethical concerns associated with direct-to-consumer genetic testing. Of further note, the business model of most direct-to-consumer genetic testing providers focuses on building large databases of genomes.^22^ The data generated are invaluable for research and serve to benefit population health through understanding how genetic factors influence disease predisposition.^23^ Such research also has the potential to improve diagnostics,^24^ develop sound prevention and treatment strategies^25^ and advance genetic discoveries and drug development.^26^

One of the most prominent controversies about direct-to-consumer genetic testing is the regulation of the field. Two central issues in regulating direct-to-consumer genetic testing are the need to protect consumers from harm and ensuring that tests are analytically and clinically valid.^27^ Many academics worldwide have claimed that direct-to-consumer genetic testing is unregulated in their jurisdictions and have advocated for legal intervention.^28^ There is limited South African literature on direct-to-consumer genetic testing, and the little that exists asserts that direct-to-consumer genetic testing is unregulated.^29^ In this article we challenge this assertion. We focus on the very first step in the direct-to-consumer genetic testing process, namely the collection of saliva samples by consumers themselves. We show that this is indeed regulated – even over-regulated – but in an unsatisfactory manner that calls for legal reform.

Here is a roadmap of our article: First, we examine the statutory scheme relevant to the collection of saliva samples by consumers as part of the direct-to-consumer genetic testing process. We review the legislation relevant to such a process and the kinds of objects regulated by the statutory scheme, namely saliva, and whether saliva falls within the statutory definitions of “tissue” and/or “biological material”. This section concludes that on a plain reading of the statutory scheme, there is an apparent ban on individuals collecting their own saliva samples for direct-to-consumer genetic testing. Next, we consider three possible arguments against the apparent ban on persons collecting their own saliva samples: (1) the ban can be overlooked based on the *de minimis* principle; (2) the purpose of the legislation is not to regulate the collection of saliva; and (3) personal autonomy. We conclude that the apparent ban on individuals collecting their own saliva samples for direct-to-consumer genetic testing is both irreconcilable with a purposive interpretation of the statutory scheme and unconstitutional. We conclude with recommendations for statutory reform.

## 2 The statutory scheme

### 2.1 Overview

The topics of health, genetic testing, and genetic research are regulated by the *National Health Act* 61 of 2003 (hereafter the *NHA*) and its relevant regulations, including the *Regulations Relating to the Use of Human Biological Material* (hereafter the *Human Biological Material Regulations*),^30^ and the *Regulations Relating to the Taking of Buccal Sample or Withdrawal of Blood from a Living Person for Testing (Amendment)* (hereafter the *Buccal Sample and Blood Withdrawal Regulations*).^31^ We refer to these statutes collectively as the “statutory scheme”.

### 2.2 The kinds of objects that are regulated by the statutory scheme

To determine whether a specific act performed in the context of genetic testing and/or genetic research is regulated by the statutory scheme depends on whether the object of the act – in this case saliva – falls within the definitional scope of the kinds of objects that are regulated by the statutory scheme (we refer to objects of this kind as “regulated objects”). This is not always a straightforward exercise, given the bifurcated nature of the lexicons used by the statutory scheme.^32^ While the *NHA* itself and some of its regulations use terms such as “tissue”, “gametes”, “blood”, and “blood products” to denote the kinds of objects that are regulated, one of the most consequential sets of regulations made in terms of the *NHA* – the *Human Biological Material Regulations* – introduces a shift away from these terms and instead uses the term “biological material”. Importantly, as we discuss in more detail below, although there is an overlap in meaning between some of the terms of the two lexicons, the terms and their meanings in the *NHA* and the *Human Biological Material Regulations* are not the same. Accordingly, in order to determine whether saliva falls within the definitional scope of the kinds of objects that are regulated by the statutory scheme, it is necessary to consider the relevant terms in each of these lexicons. First, consider the *NHA*’s term “tissue”. The *NHA* defines “tissue” as:

[H]uman tissue, and includes flesh, bone, a gland, an organ, skin, bone marrow or body fluid, but excludes blood or a gamete.^33^

Although human tissue in its common meaning does not include saliva, the fact that the *NHA*’s definition includes “body fluid” expands “tissue” beyond its normal meaning to include saliva.^34^

Second, “biological material” in the *Human Biological Material Regulations* is defined as follows:

[M]aterial from a human being including DNA, RNA, blastomeres, polar bodies, cultured cells, embryos, gametes, progenitor stem cells, small tissue biopsies and growth factors from the same.^35^

The use of the word “including” indicates that this definition is not a closed list and may incorporate other types of material from a human being.^36^ Since saliva is “material from a human being”, saliva is included in the definition of “biological material”. Accordingly, irrespective of the lexicon, saliva falls within the definitional scope of the regulated objects.

In the following section we analyse the way in which the statutory scheme regulates acts with regulated objects in the context of genetic testing and/or genetic research.

### 2.3 The apparent ban on persons who collect buccal swabs themselves

Generally, when dealing with regulated objects, there are defined categories of persons (usually qualified professionals) who are legally permitted to perform certain acts with these objects. The most apparent reason is that regulated objects are typically used in the clinical healthcare and health research contexts. Direct-to-consumer genetic testing differs in this regard as consumers (usually individual laypersons) are responsible for collecting their own saliva samples, typically with no professional oversight.^37^ Ostensibly to minimise potential harm to individuals due to the erroneous handling of samples, South Africa’s extant law appears to place the duties relating to samples on those with some sort of professional qualification. For example, the *NHA* requires a registered medical practitioner or dentist to remove, use or transplant tissue from a living person.^38^ The *Human Biological Material Regulations* permit only “competent persons” – a term that is defined to include only certain types of healthcare professionals – to undertake activities relating to biological material.^39^ But what is the position in the context of direct-to-consumer genetic testing, where it is not a professional but rather an “unqualified” consumer without specialist training who is responsible for collecting a saliva sample? Are these consumers legally permitted to collect their own saliva samples, where they are to be used in direct-to-consumer genetic testing and possibly for subsequent genetic research?

Section 59(1) of the *NHA* provides that only registered medical practitioners or dentists are permitted *inter alia* to “remove any tissue from a living person”.^40^ This clearly exclude laypersons. Similarly the *Human Biological Material Regulations* provide that only a “competent person” may remove biological material for “genetic testing, genetic health research or therapeutic purposes”,^41^ and that this must be undertaken at an authorised and prescribed institution.^42^ The definition of “competent person” in the *Human Biological Material Regulations* provides various categories under which a competent person may fall, depending on the circumstance, the activity and the purpose for which the biological material will be used.^43^ For example, a person registered as a medical practitioner or a health professional trained as a phlebotomist and registered in terms of the *Health Professions Act* 56 of 1974 (hereafter the *Health Professions Act*) or as a nurse in terms of the *Nursing Act* 33 of 2005 (hereafter the *Nursing Act*) is required for intravenous blood withdrawal,^44^ a medical practitioner registered under the *Health Professions Act* as a specialist in the procedure is required for the intra-arterial withdrawal of blood,^45^ and a urologist registered in terms of the *Health Professions Act* or a male reproductive health expert is required for the withdrawal of sperm.^46^ However, prior to listing these categories the definition states that “‘competent person’ means trained…”.^47^ This implies that a “competent person” requires some type of professional training in a particular area and thus appears to exclude laypersons, who generally lack medical or scientific expertise.

The *Buccal Sample and Blood Withdrawal Regulations* provide that a healthcare provider or a person considered in section 56 of the *NHA* who is not a healthcare provider but who has undergone specified training may take a buccal sample or remove blood from another living person.^48^ As such, the *Buccal Sample and Blood Withdrawal Regulations* are not applicable to taking a buccal sample from oneself.

A brief excursus on regulation 2 of the *Buccal Sample and Blood Withdrawal Regulations* is necessary to point out a legal anomaly. It refers to a person considered in section 56 of the *NHA* as possibly not a healthcare provider. However, this can never be the case. Section 56 of the *NHA* provides *inter alia* that a “person” may use tissue removed from a living person only for such medical or dental purposes as may be prescribed through regulation. This section in isolation does not seem to limit the kind of person that may use tissue as prescribed. However, section 59(1) of the *NHA*, referred to above, makes it clear that for the purposes of Chapter 8 of the *NHA*, which includes section 56, only a registered medical practitioner or dentist may remove any tissue from a living person. Accordingly, the “person” referred to in section 56 of the *NHA* must be a registered medical practitioner or dentist. This considered, the reference in the *Buccal Sample and Blood Withdrawal Regulations* to a person considered in section 56 of the *NHA* who is not a healthcare provider is anomalous. The *NHA* defines a “health care provider” as “a person providing health services in terms of any law”, including *inter alia* the *Health Professions Act*.^49^ Since a registered medical practitioner or dentist is a healthcare provider, the person referred to in section 56 of the *NHA* can only be a healthcare provider.

To conclude our analysis of how the statutory scheme regulates the collection of saliva for direct-to-consumer genetic testing: Both the *NHA* and the *Human Biological Material Regulations* render it unlawful for persons to collect a buccal sample themselves. Is this the writing on the wall for direct-to-consumer genetic testing?

In the following section, we analyse three possible arguments against the conclusion that persons are legally banned from collecting their own saliva samples for direct-to-consumer genetic testing.

## 3 Arguments against the apparent ban on persons collecting buccal swabs themselves

### 3.1 The ban can be ignored based on the de minimis principle

The first possible argument would be that the ban on individuals collecting their own saliva samples can be ignored based on the maxim *de minimis non curat lex* (the law does not concern itself with trifles) – also known as the *de minimis* principle.^50^ Is a seemingly harmless buccal swab something that the law should concern itself with? The problem with reliance on the *de minimis* principle is the subjectivity involved in deciding whether an issue is actually trifling or not. Those who oppose direct-to-consumer genetic testing because of ethical concerns, such as whether test results are useful and whether consumers can understand the genetic information that is provided to them without professional assistance, would argue that the statutory ban on collecting one’s own saliva sample plays an important role in protecting persons from unscrupulous direct-to-consumer genetic testing providers that function outside of the traditional healthcare system. We do not suggest that such an argument will necessarily have force in a court of law – but it could. Accordingly, it would be cavalier of direct-to-consumer genetic testing providers that consider entering the market and offering their products and services in South Africa to assume that this issue is unimportant. Therefore, the argument based on the *de minimis* principle does not contribute much to legal certainty.

### 3.2 The purpose of the legislation is not to regulate saliva

Although a plain reading of the statutory scheme renders it unlawful to collect one’s own saliva for a direct-to-consumer genetic test, in the light of South Africa’s commitment to purposive interpretation the question must be asked: Is this truly the purpose of the statutory scheme? We suggest that there are a number of indicia that point to a negative answer. First, while blood, gametes, ova, and sperm are specifically catered for in the definition of “competent person” in the *Human Biological Material Regulations*, saliva is not mentioned.^51^ One can argue that this omission indicates that the purpose of the *Human Biological Material Regulations* is not to regulate the collection of saliva. It can be countered that the *Human Biological Material Regulations* do contain a general provision that “[n]o person, except a competent person, may remove biological material”.^52^ However, lacking any provision about who would be a “competent person” in the case of saliva, this general provision does not seem to compensate for the omission in the definition of “competent person” in the *Human Biological Material Regulations*.

Second, the ostensible purpose of the provisions in the statutory scheme that only certain healthcare professionals may remove regulated objects from living persons is to protect the health and bodily integrity of persons from whom biological material is removed. But clearly there is a difference in the risk involved in the withdrawing of a vial of blood for genetic testing or research and collecting a saliva sample. While using a blood sample is common in genetic testing in the clinical context, direct-to-consumer genetic testing uses less invasive saliva samples, which consumers can easily collect at home. Although there are risks involved in collecting saliva samples, such as the contamination of the saliva sample^53^ or the compromising of DNA quality and yield^54^ leading to inaccurate results, these are not risks to the health and bodily integrity of the person providing the saliva sample for direct-to-consumer genetic testing. Collecting a saliva sample for a direct-to-consumer genetic test merely entails spitting into a tube or swabbing the inside of one’s cheek to obtain the necessary cells from which DNA can be extracted, sequenced, and analysed.^55^ By contrast, blood withdrawal involves inserting a needle into a vein, which requires precision and some medical knowledge. This poses a risk to one’s health and bodily integrity. It is evident that professional training and skill are necessary for genetic testing using blood, while collecting a saliva sample is a relatively straightforward process. In addition, direct-to-consumer genetic testing providers typically provide step-by-step instructions on how to collect a saliva sample which, if followed, should mean that there are no complications.

The third indicium that the purpose of the statutory scheme cannot be to regulate the self-collection of saliva is the practical reality that thousands of diabetics regularly prick their own fingers to check and manage their blood sugar levels – in apparent disregard of the statutory scheme. The *Human Biological Material Regulations* specifically provide for situations where a finger prick to obtain a drop of blood is sufficient for testing and, in such a case, define a “competent person” as someone registered in terms of the *Health Professions Act*.^56^ Such a “competent person” may obtain a drop of blood for “therapeutic purposes”,^57^ which would include managing a diabetic’s blood sugar levels. Does this mean that all diabetic individuals who are pricking their own fingers are contravening the *Human Biological Material Regulations*? On a plain reading of the *Human Biological Material Regulations*, the answer must be affirmative, constituting a mass-scale, ongoing contravention. However, it would be absurd to criminally prosecute all of these diabetics.^58^ Accordingly, a reading of the statutory scheme that excludes pricking one’s own finger to obtain a blood sample for therapeutic purposes is necessary. If this conclusion is accepted, then consistency would demand that the same applies to collecting one’s own saliva sample. This is amplified by considering that in the case of diabetics, a finger prick can be painful and may cause bleeding or bruising, and yet individuals do this themselves at home. By contrast, collecting a saliva sample involves no pain and should not have any side effects. In comparison to other procedures, such as blood withdrawal, the risks associated with the collection of a saliva sample are minimal and thus it can be done without professional oversight.

Given the three indicia analysed above, we suggest that a purposive interpretation of the statutory scheme points away from a prohibition on persons collecting their own saliva samples for direct-to-consumer genetic testing.

### 3.3 Personal autonomy

A value that is relevant to the current analysis is autonomy. Autonomy refers to individual freedom or self-determination.^59^ The *Stanford Encyclopedia of Philosophy* defines autonomy as:

[A]n idea that is generally understood to refer to the capacity to be one’s own person, to live one’s life according to reasons and motives that are taken as one’s own and not the product of manipulative or distorting external forces.^60^

The Constitutional Court in *Barkhuizen v Napier* (hereafter *Barkhuizen*) described “self-autonomy”^61^ as “the ability to regulate one’s own affairs, even to one’s own detriment”. In South African case law, autonomy has been recognised as a constitutional value that underlies various rights such as human dignity, freedom and privacy. In *NM v Smith* (hereafter *NM*)^62^ O’Regan J in her dissenting judgment held as follows:

Recognising the role of freedom of expression in asserting the moral autonomy of individuals demonstrates the close links between freedom of expression and other constitutional rights such as human dignity, privacy and freedom. Underlying all these constitutional rights is the constitutional celebration of the possibility of morally autonomous human beings independently able to form opinions and act on them … Our Constitution seeks to assert and promote the autonomy of individuals …^63^

From an individual’s perspective, the purpose of participating in direct-to-consumer genetic testing is to obtain information on, and insight into, one’s own genetic make-up. The self-collection of a saliva sample by an individual is the first step toward this purpose, and is done of the individual’s own free will.^64^ We therefore suggest that collecting one’s own saliva sample for direct-to-consumer genetic testing is an expression of autonomy.

The value of autonomy as well as the constitutional rights linked thereto are relevant to direct-to-consumer genetic testing in several ways. Below we analyse the constitutional rights of human dignity, privacy, and bodily integrity and show how – because each of these rights is infused by the value of autonomy – prohibiting individuals from collecting their own saliva samples for direct-to-consumer genetic testing infringes on autonomy and is thereby a violation of these constitutional rights.

First, dignity is explicitly recognised as a right in the *Constitution of the Republic of South Africa*, 1996 (hereafter the *Constitution*).^65^ In *National Coalition for Gay and Lesbian Equality v Minister of Justice* (hereafter *National Coalition*),^66^ it was held that at a minimum dignity necessitates acknowledging “the value and worth of all individuals as members of our society”.^67^ In *Teddy Bear Clinic for Abused Children v Minister of Justice and Constitutional Development* (hereafter *Teddy Bear Clinic*),^68^ the Constitutional Court linked this recognition of individual worth with respecting individual choices. The Court held that:

[D]ignity recognises the inherent worth of all individuals (including children) as members of our society, *as well as the value of the choices that they make*.^69^

In *Barkhuizen* the Constitutional Court held that autonomy is “the very essence of freedom and a vital part of dignity”.^70^ This is elaborated on by the Constitutional Court in *MEC for Education: KwaZulu-Natal v Pillay* (hereafter *Pillay*)^71^ as follows:

A necessary element of freedom and of dignity of any individual is an ‘entitlement to respect for the unique set of ends that the individual pursues’ … That we choose voluntarily rather than through a feeling of obligation only enhances the significance of a practice to our autonomy, our identity and our dignity.^72^

Accordingly, dignity demands that individuals’ autonomy be respected. We have already established that collecting one’s own saliva sample for direct-to-consumer genetic testing is an expression of autonomy. Therefore, it follows that collecting one’s own saliva sample for direct-to-consumer genetic testing is protected by the right to dignity. The apparent ban in the statutory scheme on individuals collecting their own saliva samples therefore infringes on the right to dignity.

Second, the constitutional right to privacy^73^ also shares links with autonomy and dignity. The right to privacy entails that individuals “have a right to a sphere of intimacy and autonomy that should be protected from invasion”.^74^ In *Bernstein v Bester* (hereafter *Bernstein*)^75^ Ackermann J referred to privacy as the “inner sanctum of a person”.^76^ The right to privacy includes the right to live life, within a personal realm, as one pleases,^77^ and free from interference.^78^ We suggest that an individual’s choice to participate in direct-to-consumer genetic testing and to self-collect saliva for this purpose falls within this “inner sanctum” and personal realm – and is thus protected by the right to privacy. The apparent ban on individuals collecting their own saliva sample therefore not only infringes on the right to dignity but also on the right to privacy.

Third, individuals enjoy the right to bodily integrity,^79^ which consists of two elements: “security in” and “control over” one’s body.^80^ The former represents protecting bodily integrity against external intrusions, while the latter indicates the protection of “bodily autonomy or self-determination against interference”.^81^ We suggest that the act of collecting one’s own saliva from one’s own body for direct-to-consumer genetic testing is protected by the right to bodily integrity. This adds a third rights infringement to the apparent ban on individuals collecting their own saliva sample.

In order to promote autonomy, individuals should have the freedom and the privacy to do as they wish with their own bodies, free from governmental control, and where the risk of harm to themselves and others is minimal. In line with this, prohibiting individuals from collecting their own saliva samples for direct-to-consumer genetic testing violates their autonomy. Can there be a legitimate government purpose that is served by outlawing individuals from collecting their own saliva samples? The ostensible purpose of the provisions in the statutory scheme that only certain healthcare professionals may remove regulated objects from living persons is to protect the health and bodily integrity of persons from whom biological material is removed. This is indeed a legitimate government purpose. But, as we have already argued, it is certainly not applicable to the self-collection of a saliva sample. While there are several concerns pertaining to direct-to-consumer genetic testing in the absence of a healthcare professional, we suggest that there is simply no conceivable legitimate government purpose that is served by prohibiting individuals from collecting their own saliva samples. Accordingly the relevant provisions of the statutory scheme that purport to ban individuals from collecting their own saliva samples for direct-to-consumer genetic testing are unconstitutional and hence invalid.

## 4 Conclusion

Although a plain reading of the statutory scheme prohibits individuals from collecting their own saliva samples for direct-to-consumer genetic testing, we suggest that a plain reading is evidently untenable. Not only is a plain reading incompatible with a purposive interpretation of the statutory scheme, but it is also unconstitutional. What is the impact of this conclusion on the practice of direct-to-consumer genetic testing? Simply that the first step in the process – collecting saliva samples by the consumers themselves – is lawful.

It is interesting to note that the *Draft Regulations Regarding the Use of Human DNA, RNA Cultured Cells, Stem Cells, Blastomeres, Polar Bodies, Embryos, Embryonic Tissue and Small Tissue Biopsies for Diagnostic Testing, Health Research and Therapeutics* (hereafter the *Draft Testing and Research Regulations*)^82^ – although never made into law in its draft form – explicitly provides for individuals to collect their own saliva samples. The *Draft Testing and Research Regulations* states that a “competent person” in the case of collecting cells from the inside of the cheek (buccal swab) is:

[A]ny person who has been trained to perform such a procedure *or the person himself/herself who provides the sample for genetic testing* [own emphasis].^83^

However, even if this definition of “competent person” in the *Draft Testing and Research Regulations* had become law, it would have been in ostensible conflict with a plain reading of the *NHA* and the *Human Biological Material Regulations*. As such, we recommend that the relevant provisions of the *NHA* and the *Human Biological Material Regulations* be amended to accommodate situations wherein individuals collect their own saliva (and blood by finger pricking) – aligned with the *Draft Testing and Research Regulations*.

Although the analysis in this article was limited to only the first step of the process of direct-to-consumer genetic testing, it is already evident that the assertion in the literature that direct-to-consumer genetic testing is “unregulated” in South Africa is mistaken. The first step in the process of direct-to-consumer genetic testing is regulated, but poorly so, and is in dire need of reform. The subsequent steps in the direct-to-consumer genetic testing process remain a greenfield – awaiting legal analysis.

## Footnotes

1 In 2003 the Human Genome Project published the complete sequence of the human genome, which was proclaimed to transform medicine and assist in understanding disease. Bair 2005 *Food & Drug LJ* 413.

2 Jeong 2018 *Health Policy and Technology* 233; Apathy *et al* 2018 *Am J Prev Med* 806.

3 Sarin 2015 *J Can Res Ther* 1.

4 Tiller and Lacaze 2018 *Front Public Health* 1; Howard *et al* “Convergence of Direct-to-Consumer Genetic Testing Companies and Biobanking Activities” 60; Sarin 2015 *J Can Res Ther* 1.

5 Howard *et al* “Convergence of Direct-to-Consumer Genetic Testing Companies and Biobanking Activities” 60. Also see Majumder, Guerrini and McGuire 2021 *Annu Rev Med* 153.

6 Mainetti *et al* “Usability Testing of Two Mini-Games and One Serious Game to Educate People About Genetics” 82.

7 Prainsack 2011 *ÖZP* 401.

8 Tiller and Lacaze 2018 *Front Public Health* 1.

9 Borry, Cornel and Howard 2010 *J Community Genet* 102.

10 An example of a direct-to-consumer genetic testing provider that offers such testing is 23andMe. Howard *et al* “Convergence of Direct-to-Consumer Genetic Testing Companies and Biobanking Activities” 60.

11 Allyse 2018 *Symposium on Precision Medicine* 115; Prainsack 2011 *ÖZP* 401.

12 Curnutte 2012 *Medic* 140; Helgason and Stefánsson 2010 *Dialogues Clin Neurosci* 65.

13 Jackson, Goldsmith and Skirton 2014 *Family Pract* 348.

14 Hogarth and Saukko 2017 *New Genet Soc* 198.

15 Oliveri and Pravettoni 2016 *Biol Med* 1.

16 Dandara *et al* 2013 *S Afr Med J* 511; Oh 2019 *Genomics Inform* 1.

17 Dandara *et al* 2013 *S Afr Med J* 511; Oh 2019 *Genomics Inform* 1.

18 Sweeny and Legg 2011 *Psychology and Health* 1260; Berg and Fryer-Edwards 2008 *J Bus Ethics* 19.

19 Genetic literacy denotes an individual’s basic knowledge of genetic science. It includes central genetic concepts like gene expression, transmission, and elementary awareness of how genes affect health. Pearson and Liu-Thompkins 2012 *J Public Policy Mark* 43; Field, Krokosky and Terry 2010 *Genetic Alliance* 731; Varga 2012 *J Genet Counsel* 385.

20 Lynch *et al* 2011 *J Genet Counsel* 487; Chow-White *et al* 2015 *Ethics Inf Technol* 190; Laestadius, Rich and Auer 2017 *Genet Med* 513.

21 Editorial 2012 *Lancet* 76.

22 Robertson 2009 *Berkeley Tech LJ* 218; Cacchio 2018 *UMKC L Rev* 224.

23 Dandara *et al* 2013 *S Afr Med J* 510.

24 Salloum *et al* 2018 *BMC Public Health* 1; Mattick *et al* 2014 *Med J Aust* 17; National Human Genome Research Institute 2020 https://www.genome.gov/about-genomics/fact-sheets/A-Brief-Guide-to-Genomics

25 Salloum *et al* 2018 *BMC Public Health* 1; Mattick *et al* 2014 *Med J Aust* 17; National Human Genome Research Institute 2020 https://www.genome.gov/about-genomics/fact-sheets/A-Brief-Guide-to-Genomics.

26 Robertson 2009 *Berkeley Tech LJ* 218; Cacchio 2018 *UMKC L Rev* 224; Staunton and Moodley 2016 *S Afr Med J* 136.

27 Analytical validity refers to a test’s accuracy in identifying the presence or absence of a specific genetic variant and is often dependent on the quality of the laboratory conducting the test. Clinical validity denotes a test’s ability to differentiate between individuals who possess or will develop a condition, and those who will not. It measures the accuracy of a test in detecting or predicting disease risk. While analytical validity and clinical validity involve the procedure and reliability of genetic tests, clinical utility concerns the application of such tests and examines whether they can offer information regarding the diagnosis, management, treatment or prevention of disease. Clinical utility refers to the probability of test results improving patient outcomes. Chokoshvili, Vears and Borry 2017 *Best Prac Res Cl Ob* 58; Dandara *et al* 2013 *S Afr Med J* 512; Popovsky 2010 *Dartmouth LJ* 68; Helgason and Stefánsson 2010 *Dialogues Clin Neurosci* 65; Curnutte and Testa 2012 *New Genet Soc* 167; Curnutte 2012 *Medic* 141; Hogarth, Javitt and Melzer 2008 *Annu Rev Genom Hum Genet* 178. See, also, Saunders 2010 *QMLJ* 73; Wright, Hall and Zimmern 2011 *Genet Med* 297.

28 For further information on what has been said by certain academics, see Tamir 2010 *Med Law Rev* 233–237; Tiller and Lacaze 2018 *Front Public Health* 4; Popovsky 2010 *Dartmouth LJ* 79–85; Knoppers, Avard and Howard 2010 *Expert Rev Mol Diagn* 967.

29 Only one article has been written on this topic – by Dandara *et al*., according to whom there is an “absence of regulation” of direct-to-consumer genetic testing in South Africa, and Africa as a whole. While another article on direct-to-consumer genetic testing in South Africa was written by Lombard *et al*, it focuses exclusively on whole-exome sequencing (hereafter WES). WES targets the protein-coding regions of the human genome, allowing the identification of differences in the protein-coding region of any gene – as opposed to in a few chosen genes. Direct-to-consumer genetic testing providers typically use genotyping as opposed to sequencing to analyse DNA. Genotyping determines which genetic variants are possessed by an individual. Sequencing the whole genome of an individual is not yet feasible. A South African body that mentioned direct-to-consumer genetic testing is the Academy of Science of South Africa (hereafter ASSAf), in their report *Human Genetics and Genomics in South Africa: Ethical, Legal and Social Implications* (hereafter the ASSAf Report). The ASSAf Report, while emphasising that the regulation of direct-to-consumer genetic testing is needed, does not suggest how this should be approached or achieved. The ASSAf Report merely states that “[d]irect to consumer genetic marketing and testing must be regulated”. Through the ASSAf Report’s recommendation that direct-to-consumer genetic testing be regulated, it is implied that it views the industry as currently unregulated. Dandara *et al* 2013 *S Afr Med J* 510, 512; Lombard *et al* 2016 *S Afr Med J* 139–140; 23andMe date unknown https://customercare.23andme.com/hc/enus/articles/202904600-Difference-Between-DNA-Genotyping-Sequencing; ASSAf *Human Genetics and Genomics in South Africa* 34–35, 38.

30 GN R177 in GG 35099 of 2 March 2012 (hereafter the *Human Biological Material Regulations*).

31 GN R944 in GG 34750 of 11 November 2011 (hereafter the *Buccal Sample and Blood Withdrawal Regulations*).

32 For a more detailed discussion on this issue, see Thaldar and Shozi forthcoming *SALJ*.

33 Section 1 of the *National Health Act* 61 of 2003 (the *NHA*). This is also followed by the *Regulations Regarding the General Control of Human Bodies, Tissue, Blood, Blood Products and Gametes* (GN R180 in GG 35099 of 2 March 2012) (hereafter the *General Control Regulations*).

34 “Body fluid” commonly refers to “a fluid or fluid secretion (such as blood, lymph, saliva, semen, or urine) of the body”. Merriam-Webster date unknown https://www.merriam-webster.com/medical/body%20fluid.

35 Regulation 1 of the *Human Biological Material Regulations*. While “tissue” is mentioned in this definition (although not defined), it refers specifically to “small tissue biopsies” which have a different meaning to simple “tissue”. It is interesting to note that reg 1 of the *Draft Regulations Regarding the use of Human DNA, RNA Cultured Cells, Stem Cells, Blastomeres, Polar Bodies, Embryos, Embryonic Tissue and Small Tissue Biopsies for Diagnostic Testing, Health Research and Therapeutics* (GN R7 in GG 29526 of 5 January 2007) (hereafter the *Draft Testing and Research Regulations*) defines “biological material” as “any material from a human being, including blood, cells, tissues, DNA, RNA, polar bodies, blastomeres, embryos and gametes”. Unlike the *Human Biological Material Regulations*, the *Draft Testing and Research Regulations* specifically include “tissue” in the definition of “biological material”.

36 This is also in line with the principles of statutory interpretation. See *Minister of Safety and Security v Xaba* 2003 2 SA 703 (D & CLD) 713GE-714B.

37 This is so, unless the direct-to-consumer genetic test or provider requires the involvement of a healthcare professional to collect the saliva sample on the consumer’s behalf.

38 Section 59(1) of the *NHA*.

39 Regulation 1 of the *Human Biological Material Regulations*.

40 Section 59(1) of the *NHA*.

41 Regulation 2(a) of the *Human Biological Material Regulations*.

42 Regulation 2(b) of the *Human Biological Material Regulations*.

43 Regulation 1 of the *Human Biological Material Regulations*.

44 Regulation 1 of the *Human Biological Material Regulations*.

45 Regulation 1 of the *Human Biological Material Regulations*.

46 Regulation 1 of the *Human Biological Material Regulations*. Note that the withdrawal of sperm is not necessarily through self-stimulation, but can also be a surgical procedure.

47 Regulation 1 of the *Human Biological Material Regulations*.

48 Regulation 2 of the *Buccal Sample and Blood Withdrawal Regulations*. This same provision also refers to a “buccal sample” as “cellular material inside the mouth”. Reg 4 provides that when persons who are not healthcare providers take a buccal swab, this may be done only by swabbing the inside of the cheek using specified equipment to gather “a small quantity of cellular material sufficient for testing”.

49 Section 1 of the *NHA*.

50 The *de minimis* principle permits insignificant issues to be not subject to a legal requirement. LexisNexis date unknown https://www.lexisnexis.co.uk/legal/glossary/de-minimis.

51 Regulation 1 of the *Human Biological Material Regulations*.

52 Regulation 2(a) of the *Human Biological Material Regulations*. Note that this article focusses only on the removal of saliva and not on the transportation or containment thereof. Other legal principles are applicable to the transportation, containment, exporting, processing and use of saliva. These principles will be dealt with in subsequent articles.

53 Thrush and McCaffrey 2010 *J Nurse Pract* 272.

54 23andMe date unknown https://customercare.23andme.com/hc/enus/articles/202904530-Providing-Saliva-Sample-for-DNA-Test-Kit.

55 23andMe date unknown https://www.23andme.com/howitworks/;Oh 2019 *Genomics Inform* 1.

56 Regulation 1 of the *Human Biological Material Regulations*.

57 Regulation 2(a) of the *Human Biological Material Regulations*.

58 Regulation 14 of the *Human Biological Material Regulations*, dealing with offences, states that “[a]ny person who contravenes these regulations or fails to comply with any provision of these regulations, is guilty of an offence, and liable upon conviction to a fine or imprisonment of not more than 10 years, or both such fine and such imprisonment”.

59 Jordaan 2009 *Journal of Philosophy, Science & Law* 4.

60 Christman 2020 https://plato.stanford.edu/entries/autonomy-moral/.

61 We agree with the views of Thaldar and Steytler who state that the term “self-autonomy”, as used by the Constitutional Court in *Barkhuizen v Napier* 2007 5 SA 323 (CC) (hereafter *Barkhuizen*) para 57, is an “unnecessary tautology” and does not differ from “autonomy”. Thaldar and Steytler 2021 *SALJ* 273.

62 *NM v Smith* 2007 5 SA 250 (CC) (hereafter *NM*).

63 *NM* paras 145–146. This was also cited with approval in the High Court case of *AB v Minister of Social Development* 2016 2 SA 27 (GP) paras 65–66 (hereafter *AB 1*), and by the Supreme Court of Appeal (SCA) in *British American Tobacco South Africa (Pty) Ltd v Minister of Health* 2012 3 All SA 593 (SCA) para 13.

64 In the medical context, informed consent is paramount in any decision-making process. Informed consent aims to protect against harm and promotes autonomous decision-making. However, informed consent is not always required beyond this, and individuals are assumed to simply make a decision based on their own knowledge and information that they can obtain themselves. While some have argued that direct-to-consumer genetic testing falls outside of the medical sphere, we suggest that this is clearly not the case because, although direct-to-consumer genetic testing providers offer health insights and information on predisposition to a variety of diseases and conditions, they do not purport to provide medical advice or diagnoses and recommend that consumers consult with healthcare professionals regarding their test results before making any decisions. This informs the free will and autonomy of individuals as it provides them with a choice. However, free will is impeded when direct-to-consumer genetic testing providers mislead consumers. We note concerns raised by some about misleading information on direct-to-consumer genetic testing allegedly propagated by certain direct-to-consumer genetic testing providers, which would undermine consumer autonomy. Given that direct-to-consumer genetic testing providers operate for-profit, they may exaggerate facts in advertisements and distort information on their websites in order to promote their tests and increase sales. Although consumers decide to undergo direct-to-consumer genetic tests and determine their utility, their decisions may be inappropriate if the direct-to-consumer genetic testing provider offers erroneous or misleading information. If true, this would be *contra* ss 4(5)(*b*), 24, 29, 41, and 51(1)(*a*)(*ii*) of the *Consumer Protection Act* 68 of 2008 (hereafter the *CPA*), and thus unlawful. In addition, in extreme cases of fraud there would also be common law remedies. However, our analysis in this article is based on the assumption of lawful conduct by direct-to-consumer genetic testing providers. Bunnik *et al* 2012 *Bioethics* 345; Mahmoud-Davis 2020 *Wash U Global Stud L Rev* 39; Saukko 2013 *Proc Nutr Soc* 58–59; Popovsky 2010 *Dartmouth LJ* 78; Hogarth, Javitt and Melzer 2008 *Annu Rev Genom Hum Genet* 169. Note that direct-to-consumer genetic tests do not purport to provide clinical diagnoses. In the event of genetic tests for clinical diagnosis, there are a variety of samples which may be used. However, blood samples tend to be used more commonly than saliva samples, primarily because blood tends to contain a higher volume of DNA. This information was obtained from email correspondence with Professor Michele Ramsay on 4 February 2022.

65 Section 10 of the *Constitution of the Republic of South Africa*, 1996 (the *Constitution*) states that “[e]veryone has inherent dignity and the right to have their dignity respected and protected.”

66 *National Coalition for Gay and Lesbian Equality v Minister of Justice* 1999 1 SA 6 (CC) (hereafter *National Coalition*).

67 *National Coalition* para 28.

68 *Teddy Bear Clinic for Abused Children v Minister of Justice and Constitutional Development* 2014 2 SA 168 (CC) (hereafter *Teddy Bear Clinic*).

69 *Teddy Bear Clinic* para 52 cited in *AB 1* para 89. Emphasis added.

70 *Barkhuizen* para 57.

71 *MEC for Education: Kwazulu-Natal v Pillay* 2008 1 SA 474 (CC) (hereafter *Pillay*).

72 *Pillay* para 64. This was also repeated in the High Court case of *AB 1* para 89, where Basson J expressly stated that autonomy is “a vital part of human dignity”.

73 Section 14 of the *Constitution* states that “[e]veryone has the right to privacy, which includes the right not to have –

- their person or home searched;
- their property searched;
- their possessions seized; or
- the privacy of their communications infringed”.

74 *Khumalo v Holomisa* 2002 5 SA 401 (CC) para 27. In *NM* para 131, O’Regan J also held that privacy “presupposes personal space within which to live this life”.

75 *Bernstein v Bester* 1996 2 SA 751 (CC) (hereafter *Bernstein*).

76 *Bernstein* para 67.

77 *NM* para 33.

78 *NM* para 45.

79 Section 12(2)(*b*) of the *Constitution* provides that “[e]veryone has the right to bodily and psychological integrity”, including the right to “security in and control over their body”. Bodily integrity refers to each individual’s right to self-determination and autonomy regarding their body. Individuals are entitled to make decisions regarding their own bodies, free from unwarranted involvement by others. Nienaber and Bailey 2016 *SAJBL* 74; CRIN 2018 https://archive.crin.org/en/home/what-wedo/policy/bodily-integrity.html.

80 Section 12(2)(*b*) of the *Constitution*.

81 Currie and De Waal *Bill of Rights Handbook* 287. Also see Nienaber and Bailey 2016 *SAJBL* 74.

82 GN R7 in GG 29526 of 5 January 2007.

83 Regulation 1 of the *Draft Testing and Research Regulations*. Emphasis added. However, the provisions of the *Draft Testing and Research Regulations* do not mention a competent person in such a situation. Besides the definition, the only other mention of “competent person” in the *Draft Testing and Research Regulations* is in reference to using stem cells for therapeutic cloning. Reg 11 of the *Draft Testing and Research Regulations*.

## List of Abbreviations

Am J Prev Med: American Journal of Preventative Medicine
Annu Rev Genom Hum: Annual Review of Genomics and Human
Genet: Genetics
Annu Rev Med: Annual Review of Medicine
ASSAf: Academy of Science of South Africa
Berkeley Tech LJ: Berkeley Technology Law Journal
Best Prac Res Cl Ob: Best Practice & Research: Clinical Obstetrics & Gynaecology
Biol Med: Experimental Biology and Medicine
CPA: Consumer Protection Act 68 of 2008
CRIN: Children Rights International Network
Dartmouth LJ: Dartmouth Law Journal
Dialogues Clin Neurosci: Dialogues in Clinical Neuroscience
DNA: Deoxyribonucleic acid
Ethics Inf Technol: Ethics and Information Technology
Expert Rev Mol Diagn: Expert Review of Molecular Diagnostics
Family Pract: Family Practice
Food & Drug LJ: Food and Drug Law Journal
Front Public Health: Frontiers in Public Health
Genomics Inform: Genomics & Informatics
Genet Med: Genetics in Medicine
J Bus Ethics: Journal of Business Ethics
J Can Res Ther: Journal of Cancer Research and Therapeutics
J Community Genet: Journal of Community Genetics
J Genet Counsel: Journal of Genetic Counseling
J Nurse Pract: Journal for Nurse Practitioners
J Public Policy Mark: Journal of Public Policy and Marketing
Med J Aust: The Medical Journal of Australia
Med Law Rev: Medical Law Review
New Genet Soc: New Genetics and Society
NHA: National Health Act 61 of 2003
ÖZP: Osterreichische Zeitschrift für Politikwissenschaft
Proc Nutr Soc: Proceedings of the Nutrition Society
QMLJ: Queen Mary Law Journal
S Afr Med J: South African Medical Journal
SAJBL: South African Journal of Bioethics and Law
SALJ: South African Law Journal
UMKC L Rev: UMKC Law Review
Wash U Global Stud L: Washington University Global Studies
Rev: Law Review
WES: Whole-Exome Sequencing
